# Carpet Grass Polyphenol Reductive Degradation of Aqueous Nitrate—A Conceptual Field Application Study

**DOI:** 10.1021/acsomega.4c06522

**Published:** 2024-11-14

**Authors:** Chenju Liang, Wei-Sin Tao, Chia-Lu Shih, Yi-Wun Ye, Chun-Ting Li, Chi-Wei Wang

**Affiliations:** † Department of Environmental Engineering, National Chung Hsing University, 145 Xingda Road, Taichung 402 402202, Taiwan; ‡ Department of Environmental Engineering, Da-Yeh University, 168 University Road, Dacun, Changhua 515006, Taiwan

## Abstract

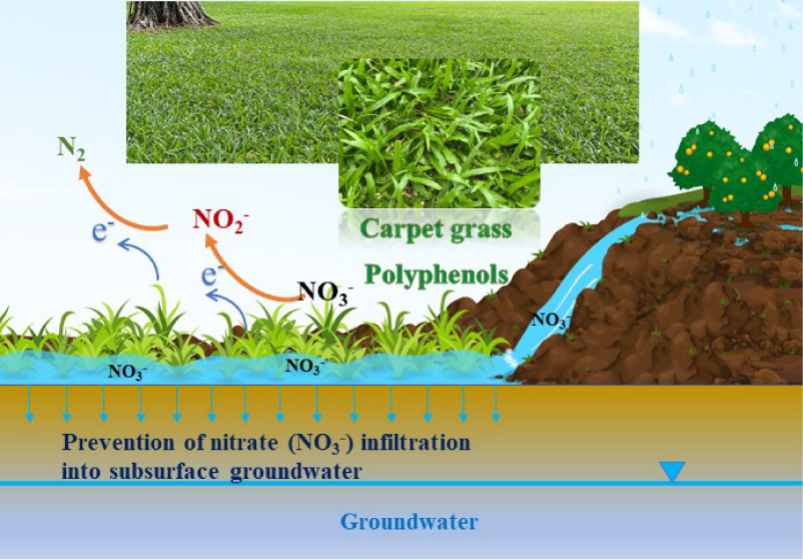

Rainwater flowing along the ground, and from hard surface such as pavement and roofs, becomes surface water runoff, which flows to surface waters, and infiltrates into the ground to become groundwater. Surface water runoff can contain elevated levels of nitrates (NO_3_^–^) from various sources including animal wastes and fertilizers. Reducing elevated levels of NO_3_^–^ in surface water runoff can minimize and/or prevent groundwater and surface water contamination. Natural polyphenols in carpet grass, due to phenolic hydroxyl groups, can degrade aqueous NO_3_^–^. This study evaluated the potential for carpet grass to purify water by denitrifying NO_3_^–^ as surface water flows through grass-covered land. The research investigated nitrate removal efficiency and reaction kinetics under various flow rates and doses of carpet grass, validating the feasibility of using natural polyphenols for water purification. A grass dose of 100 g and a retention time of 24 h, which produced approximately 20–80 mg/L as gallic acid equivalent (GAE) of polyphenol in simulated surface water runoff, were demonstrated to effectively degrade NO_3_^–^ in aqueous solution (110 mg/L) and result in the denitrification of NO_3_^–^ to nitrite (NO_2_^–^) and eventually N_2_. The first-order reaction kinetic rate constants for NO_3_^–^ degradation, NO_2_^–^ formation, and subsequent degradation of NO_2_^–^ are *k*_obs (NO3-degradation)_ = 6.89 × 10^–2^ h^–1^, *k*_1(NO2-formation)_ = 5.11 × 10^–2^ h^–1^, and *k*_2(NO2-degradation)_ = 4.63 × 10^–2^ h^–1^, respectively, with a conversion rate (α) of NO_3_^–^ to NO_2_^–^ to be 0.74. Implementing natural vegetation, such as carpet grass, in water management practices offers an environmentally sustainable approach to reducing nitrate contamination in surface water runoff.

## 1 Introduction

Groundwater serves as a crucial water source for industrial, agricultural, and domestic purposes. However, according to Hosono et al.,^1^ agricultural activities are identified as a significant contributor to nitrate (NO_3_^–^) contamination in groundwater. Excessive ingestion of NO_3_^–^ can lead to health risks such as methemoglobinemia (blue baby syndrome) and organ hypoxia.^2,3^ Furthermore, nitrate pollution can have adverse environmental effects, including toxicity to mosses, indirectly impacting biodiversity,^4^ and causing eutrophication of lakes and rivers due to excess nitrogen and phosphorus, thereby affecting ecosystems.^5^ Consequently, various nations have established regulations to control the levels of nitrate nitrogen (NO_3_^–^-N) and nitrite nitrogen (NO_2_^–^-N) in water. Drinking water standards set by the World Health Organization^6^ and Taiwan mandate NO_3_^–^-N levels to be at 50 mg/L and 10 mg/L, respectively.^7^

In recent years, water scarcity has become a pressing issue, making groundwater a crucial source of water supply. Consequently, preventing nitrate contamination in groundwater has become a key priority. Surface water runoff infiltrating into the ground serves as a replenishment source for groundwater. Therefore, reducing nitrate in surface water runoff is one of the ways that could help prevent nitrate infiltration into groundwater. The treatment of nitrate-polluted water generally involves separation and reduction processes. Separation technologies, such as electrocoagulation and membrane filtration, aim to physically remove nitrate ions. Reductive reaction technologies, including biological denitrification and chemical reactions, focus on converting nitrate into harmless nitrogen gas.^8^

Polyphenols, abundantly found in plants, possess reducing properties and exhibit diverse beneficial effects. With over 8000 different polyphenolic compounds in nature, they share chemical similarities with phenolic substances. Polyphenols not only display potent antioxidant properties^9^ but also possess antihistaminic, antibacterial, and anti-inflammatory properties.^10^ Structurally, polyphenols feature at least one hydroxyl group (OH^–^) attached to a benzene ring and can be classified into flavonoids and nonflavonoids. Nonflavonoid compounds, such as phenolic acids, stilbenes, and lignans, consist of a single aromatic ring, while flavonoids are characterized by two benzene rings connected by a three-carbon bridge and include flavones, flavonols, flavanones, anthocyanins, and isoflavones.^9^

Polyphenols possess strong reducing power due to the presence of phenolic hydroxyl groups in their structures,^11^ with the extent of their reducing power determined by the abundance and arrangement of these hydroxyl groups. Polyphenols suitable for use as antioxidants must meet two fundamental criteria: if the concentration of polyphenols is lower than that of the substrate to be oxidized, they can prevent auto-oxidation or free radical-mediated oxidation; additionally, the intermediate radicals formed after scavenging must be stabilized through further oxidation via intramolecular hydrogen bonding.^12^ Taking the structure of catechol (Ph(OH)_2_) in polyphenol molecules as an example (associated Gibbs free energy for selected compounds are presented in Table S1), the reaction equation for the release of electrons is represented as eq 1):^13^
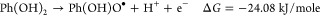
1

The half-reaction equation for the reduction of nitrate is represented as eq 2):^14^
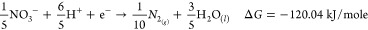
2

Based on the known properties of polyphenols and their ability to reduce nitrate, a plausible reaction mechanism for the degradation of nitrate by polyphenols can be represented by eq 3):
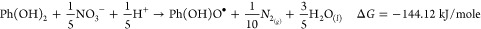
3

**Figure 1:**
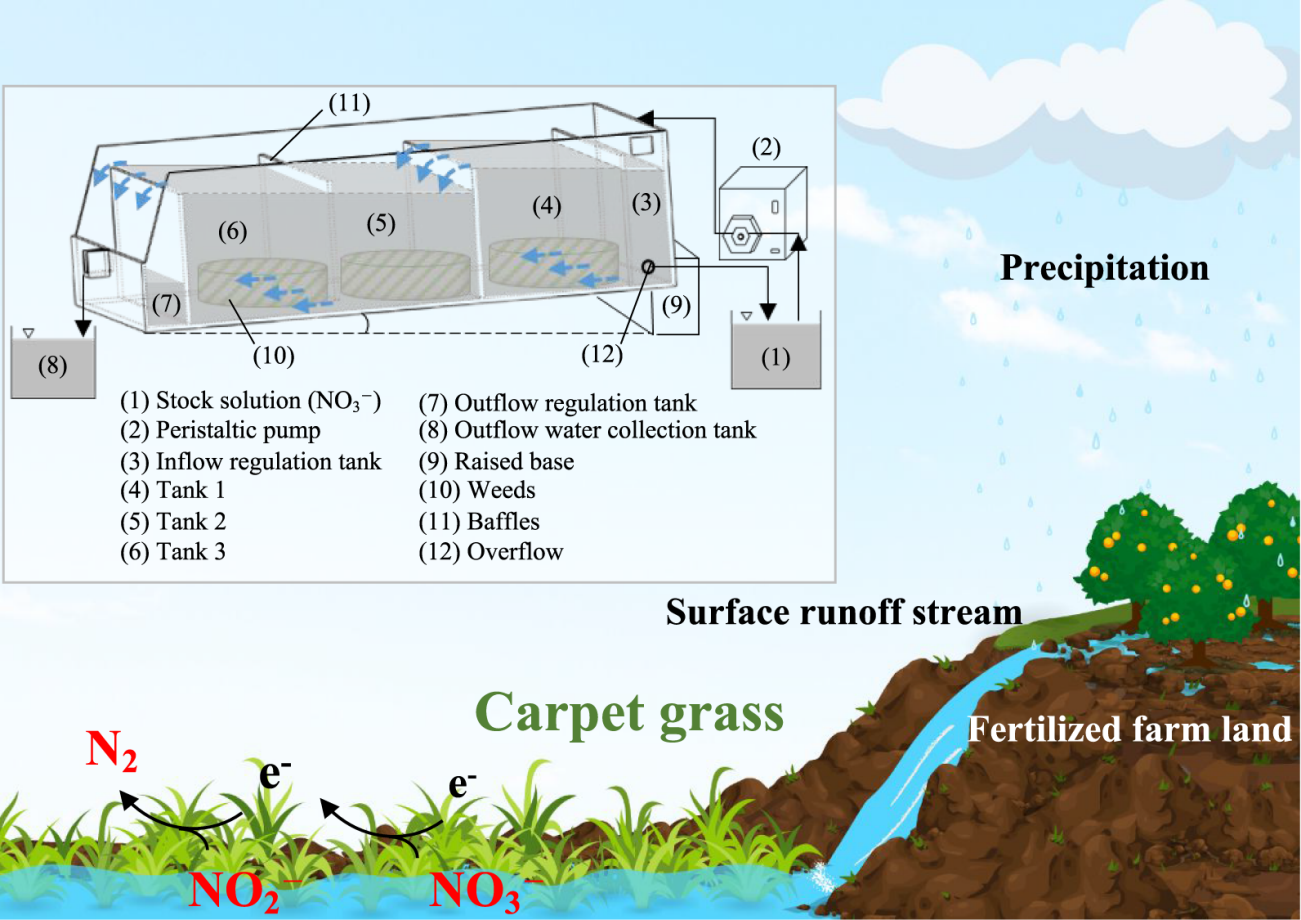
Scenario depicting surface water runoff containing nitrate flowing through carpet grass, and being purified by natural polyphenols. Inset illustrating the experimental setup designed to simulate this purification scenario.

Based on the results of the free energy calculation, it is speculated that the reaction of polyphenols in degrading nitrate is spontaneous. The research studies by Liang group et al. have demonstrated that natural polyphenols from sources such as tea leaves,^15^ wild herbs,^16^ and guava leaves^17^ can be used to degrade organic pollutants containing chlorine and nitrogen. In this study, carpet grass, a perennial herbaceous plant of the *Poaceae* family, was selected as a source of natural polyphenol reductants. Carpet grass spreads rapidly, forming a carpet-like surface, making it suitable for lawn construction.^18^ Its roots have soil-fixing properties, making it an excellent soil conservation plant. This study aimed to utilize the rich polyphenolic content of carpet grass to evaluate its potential to degrade nitrate contained within surface water runoff (schematic field application as shown in Figure 1). When such water flows through land covered by carpet grass vegetation, it comes into contact with the polyphenols released by the grass, leading to denitrification of nitrate according to eq 3) and thus contributing to water purification. Therefore, this study sought to investigate the removal efficiency of nitrate from water under different flow rates, and containing various doses of carpet grass, and also their reaction kinetic behaviors, aiming to validate the feasibility of natural polyphenols in degrading nitrate and preventing/minimizing nitrate contamination in surface water bodies, and groundwater.

## 2 Materials and Methods

### 2.1 Chemicals and Materials

Sodium nitrate (NaNO_3_, > 99%) was purchased from Union Chemical Works Ltd. Gallic acid (C_6_H_2_(OH)_3_COOH), Folin and Ciocalteu’s phenol reagent (2 N) were purchased from Sigma-Aldrich. Sodium carbonate (Na_2_CO_3_, 90–100%), and sulfuric acid (H_2_SO_4_, 95–97%) were purchased from Honeywell. Certified Reference Material (F^–^, Cl^–^, NO_2_^–^, Br^–^, NO_3_^–^, SO_4_^2–^, PO_4_^3–^, 1000 ppm) was purchased from CPAchem Ltd. Sodium bicarbonate (NaHCO_3_, 99.7%–100.3%) was purchased from J.T. Baker. The water used in the experiments was prepared using an Elga Micra Type II deionized (DI) water system (ELGA ET-C220929). The *Axonopus compressus* (carpet grass) was sourced from the campus of National Chung Hsing University, Taiwan. The flow tank design (see inset flow tank illustration in Figure 1, and its detailed dimensions in Table S2) was created using SolidWorks 2020 software by SolidWorks Corporation. The flow tank was fabricated using polylactic acid material with a PING DUAL 300 3D printer manufactured by Linkin Factory CO., Ltd. In all experiments, fresh carpet grass was collected on the day of the experiment and washed with RO water.

### 2.2 Experimental Procedures

The first phase of the experiment focused on evaluating the efficacy of removing nitrate (110 mg/L equivalent as 24.83 mg NO_3_^–^-N/L) from water using varying doses of carpet grass (30 and 100 g) at different water outflow rates (1 and 3 mL/min). Selected doses of carpet grass were enclosed in plastic mesh bags and placed inside the apparatus tank. Water was pumped from the stock solution tank to the inflow holding tank, and the flow rate was adjusted to achieve the desired retention time. Sampling occurred at specified intervals, as listed in Table S3, to assess water parameters within the experimental setup, including nitrate, nitrite, polyphenol content, oxidation–reduction potential (ORP), dissolved oxygen (DO), electrical conductivity (EC), and pH levels. In the second phase of the experiment, the dosages of carpet grass and outflow rates were selected according to the operational conditions of the tests that demonstrated superior nitrate removal efficiency in the first phase.

### 2.3 Analysis

Polyphenol content was determined using gallic acid as the standard, and Folin-Ciocalteau reagent.^19^ In a 20 mL glass bottle, 0.2 mL of the water sample was mixed with 5.6 mL of reagent water, then 4 mL of 2% sodium carbonate and 0.2 mL of 50% Folin-Ciocalteu reagent were added simultaneously. The mixture was shaken and allowed to stand at room temperature for 30 min, after which absorbance was measured at a wavelength of 750 nm using a spectrophotometer (HACH/DR3900). The phenolic content was expressed as mg of gallic acid equivalents (GAE). The antioxidant capacity was assessed using the conjugated diene method.^20^ Detailed antioxidant analytical procedures employed in this study can be found in Wang and Liang.^21^ All samples were analyzed in duplicate, and the average value was calculated. Nitrate and nitrite concentrations were determined using ion chromatography (AG 925, Metrohm) on an SH-AC-4 Anion Column (250 mm × 4.6 mm, SHINE). The eluent flow rate was 1.5 mL/min, with a sample injection volume of 25 μL. The analysis time was 20 min, with retention times for nitrate and nitrite peaks at approximately 10.5 and 7 min, respectively. The laboratory benchtop pH/mV/EC/TDS/salinity meter (Hanna HI5521), equipped with pH (Hanna HI1131) and conductivity electrodes (Hanna HI76312), was used to measure pH and EC. A pH/ORP/ISE meter (Hanna HI5222), equipped with an oxidation–reduction electrode (Mettler Toledo), was used to measure ORP. DO was measured using an Oxi 3310 (WTW GmbH) equipped with a CellOx 325 membrane-type galvanic oxygen electrode.

## 3 Results and Discussion

It was determined that the carpet grass contained approximately 7 mg-GAE/g-grass, which is within the range of total polyphenol content of 2–12 mg-GAE/g-weed from eight natural weeds (not including carpet grass, reported by Luong et al.^22^ Some characteristics of carpet grass are presented in Table 1. The carpet grass was determined to have an antioxidation capacity of 35% (lower than the range of 40–130% reported by Luong et al.^22^ Moreover, in the 50 g-grass/L solution, the EC of 1943 μS/cm indicated significant ionic species dissolution in the solution, and ORP decreased to −386 mV, indicating a highly reducing aqueous environment. These conditions can be attributed to the presence of polyphenols, which would release electrons upon dissociation, undergoing H^+^/e^–^ processes. Depending on the number and positions of OH on the structures of polyphenolic compounds, sequential proton loss electron transfer reaction mechanisms may proceed.^13^ As a result, DO was reduced by scavenging, with the released electrons and the dissociated hydrogen causing slightly acidic pH. It should be noted that some ionic species known to be contained in the grass were detected in the solution (as seen in Table 1). The analysis of polyphenol constituents in carpet grass conducted by Wang et al.^23^ pointed that phenolic acid compounds could be predominantly in grasses of the Poaceae family.^24^ However, the lack of commercially available standard solutions for phenolic acid compounds necessitates additional detailed investigation.

**Table 1:** Characteristics of *Axonopus compressus* (Carpet grass)^a^

Properties	Values
pH	5.9
ORP (mV)	–386
EC (μS/cm)	1943
DO (mg/L)	3.1
Antioxidation capacity (%)	35
Moisture content (%)	160 ± 3
Polyphenol content (mg-GAE/g-grass)	7.02 ± 0.58
Anion (mg_anion_/g_dry-grass_)	F^–^ 0.005; Cl^–^ 3.731; NO_3_^–^ 0.005; PO_4_^3–^ 0.082; SO_4_^2–^ 0.178

a Aqueous parameters were measured in the solution containing 50 g grass/L water soaked for 24 h within a temperature-controlled chamber at 20 °C in a dark environment. Moisture content was determined in accordance with the method (NIEA S280.61C) established by the National Environmental Research Academy.

In the first phase of experiments, two levels of grass doses and retention times within the tank were evaluated. Variations of NO_3_^–^ and associated NO_2_^–^ formation and degradation, and other monitored water parameters (DO, pH, EC, ORP, and polyphenol) are presented in Figures S1–S4. The results of control tests (i.e., NO_3_^–^ solution without grass, and water with only grass) are presented in Figures S5 and S6, respectively. During the operation of all tests, except for the test of a grass dose of 100 g and a retention time of 24 h, variations in water qualities indicated the following: NO_3_^–^ showed no changes and NO_2_^–^ was not generated, DO generally decreased to approximately 2–4 mg/L in the presence of grass, pH remained near neutral, ORP, with background values of approximately 220 mV, decreased in the presence of grass to around zero or slightly negative, EC remained at approximately 200–300 μS/cm and polyphenol ranged 2–5 mg-GAE/L in the presence of grass.

**Figure 2:**
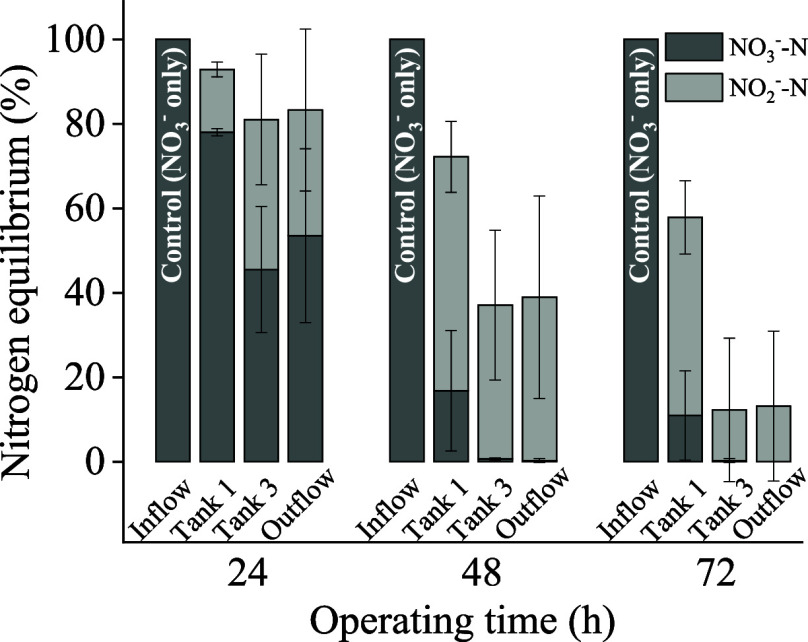
Nitrogen balance vs time showing denitrification of NO_3_^–^ to NO_2_^–^ during NO_3_^–^ solution flowing through four sampling points within the flow path, with 100 g of grass at retention time 24 h.

When the grass dose was increased to 100 g and the retention time extended to 24 h (see Figure S4), water quality parameters were comparable to those under other conditions, with the exception of elevated values for EC (500–800 μS/cm) and polyphenol (40–80 mg-GAC/L). Additionally, with increased grass dose and retention time, it was observed that NO_3_^–^ gradually decreased while NO_2_^–^ increased (see Figure S4 for NO_3_^–^ and NO_2_^–^ raw data, and Figure 2 for aqueous nitrogen balance). Over the course of 72 h, influent NO_3_^–^ rapidly decreased in Tank 1, with no NO_3_^–^ detected in the effluent. Less than 20 mg/L of NO_2_^–^ was detected in the effluent, accounting for approximately 12% of the nitrogen mass balance, with the rest speculated to be nitrogen gas leaving the system. The polyphenol content reached approximately 80 mg-GAE/L in the effluent. In summary, the gradual release of polyphenols from the grass, combined with increased NO_3_^–^ retention time in the system, can result in the denitrification of NO_3_^–^ to NO_2_^–^, and eventually N_2_, as postulated in eq. 2. To further verify this reaction, an extended reaction time under the same experimental conditions was conducted.

**Figure 3:**
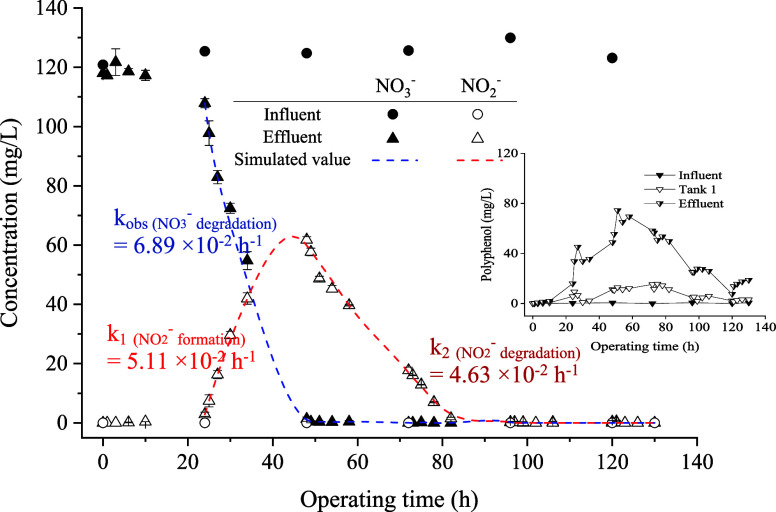
Variation of NO_3_^–^ and NO_2_^–^ vs time, in NO_3_^–^ solution flowing through the inflow, Tank 1, and outflow sampling points of the experimental flow tank, with 100 g of grass at retention time of 24 h under an extended reaction time.

To better understand the variations and trends of NO_3_^–^ to NO_2_^–^, the experiment duration was extended to 130 h, and the results are presented in Figure 3. As can be seen, a lag time of approximately 24 h of water flushing caused NO_3_^–^ degradation and NO_2_^–^ formation. The effluent NO_3_^–^ concentration after 34 h operation decreased to meet Taiwan’s regulatory standards (44.29 mg/L, equivalent as NO_3_^–^-N 10 mg/L). The NO_2_^–^ concentration started to increase at 24 h, reached a peak of 60 mg/L at 48 h, and then decreased to meet the regulatory standard (0.33 mg/L, equivalent as NO_2_^–^-N 0.1 mg/L) by approximately 96 h. The experimental results for the first 72 h were generally consistent with the results in Figure S4. Regarding polyphenol variation, it started to rise between 10 and 24 h, peaked at 74 mg/L at 50 h, and then gradually decreased to about 20 mg/L in the effluent by the end of the experiment. During the period of polyphenol concentration decline, NO_3_^–^ and NO_2_^–^ levels remained low, indicating that the polyphenol concentration was still sufficient to maintain the NO_3_^–^ and NO_2_^–^ degradation.

The reductive degradation of nitrate to intermediate nitrite, as described by eq. 4, can be effectively modeled using first-order decay kinetics. This process is mathematically represented by eq 5. Based on the data presented in Figure 3, the rate constant (*k*_obs_) for this reaction is calculated to be 6.89 × 10^–2^ h^–1^. The rate constant (*k*_1_) for the formation of NO_2_^–^ (as depicted in eq 6), accompanying the decrease of NO_3_^–^, can be described using the rate law for parallel first order formation of reaction byproducts. Subsequently, the degradation of NO_2_^–^ occurred, with the rate following eq 7 (*k*_2_ representing the rate constant). The integrated rate law for NO_2_^–^ (eq 8) characterizes the dynamic transformation of NO_3_^–^ to NO_2_^–^.^25 −.27^ The derivations for solving *k*_1_, *k*_2_, and α, as referenced from Wang et al.,^15^ involve utilizing ordinary differential equations to transform eq 7 to eq 8. Newton’s method was then employed to solve the rate constants, with the associated derivation of kinetic calculation presented in Table S4. The kinetic data were obtained, *k*_1(NO2-formation)_ = 5.11 × 10^–2^ h^–1^, *k*_2(NO2-degradation)_ = 4.63 × 10^–2^ h^–1^ and α = 0.74. Note that α is the fraction of NO_3_^–^ transformed to NO_2_^–^. Based on the calculated kinetic rate constants, the nonlinear simulated trend lines for NO_3_^–^ degradation, and subsequent NO_2_^–^ formation and degradation are illustrated in Figure 3. These trend lines show good fits with the experimental data (R^2^ = 0.994).
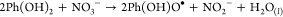
4
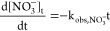
5
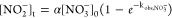
where
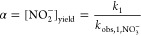
6
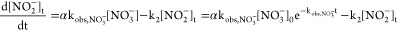
7
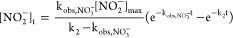
where
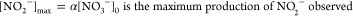
8

**Figure 4:**
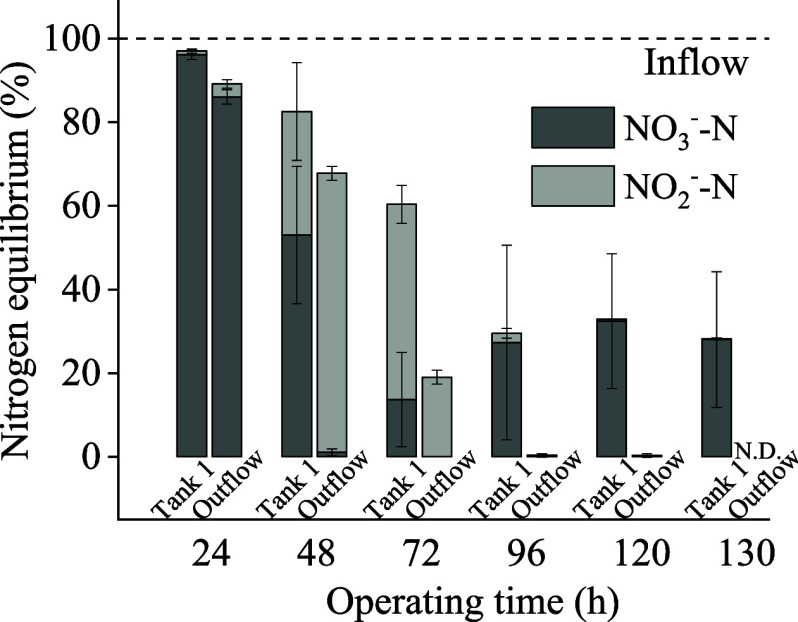
Nitrogen balance over the course of denitrification of NO_3_^–^ to NO_2_^–^ during NO_3_^–^ solution flowing through two sampling points at Tank 1 and the Outflow within the flow path, with 100 g of grass at retention time of 24 h, under an extended reaction time.

The two control experiments confirmed that no NO_3_^–^ or NO_2_^–^ was detected when grass was soaked in pure water for 72 h of operation (Figure S6), and NO_3_^–^ in solution alone did not transform into other substances (Figure S5). Therefore, it can be inferred that the significant disappearance of NO_3_^–^ between 24 and 48 h should be attributed to its transformation into NO_2_^–^, which significantly appeared during the same period. According to Figure 4, the total nitrogen mass balance remained almost unchanged from 24 to 48 h, with the percentage of nitrate nitrogen continuously decreasing, and nitrite nitrogen increasing, indicating that the main reaction during this period was the transformation of nitrate into nitrite. During the 48 to 72 h period, nitrate nitrogen was almost completely consumed, and nitrite nitrogen continued to decrease, along with the total nitrogen amount, suggesting that the primary reaction during this period was the transformation of nitrite nitrogen into nitrogen gas,^28^ hence the continuous reduction in the total nitrogen amount in the effluent.

It can be concluded that between 24 and 48 h, the release of polyphenols significantly increased, which coincided with the greatest changes in NO_3_^–^ and NO_2_^–^ concentrations. According to Amić et al.,^13^ polyphenols release electrons, enabling reduction and degradation reactions, resulting in nitrate disappearance. Therefore, the timing of polyphenol release determines the initiation time of nitrate degradation, and the amount of nitrate processed depends on the concentration and duration of polyphenol release. The experimental results may suggest that carpet grass must be soaked in water for 24 to 48 h to release significant amounts of polyphenols and start inducing reductive reactions. Further research may need to focus specifically on fractionating the polyphenols in carpet grass extract to identify the individual compounds present. However, in a related study by Wang et al.,^23^ it was demonstrated that water-extracted carpet grass releases natural polyphenols with excellent reducing power, as observed in the reductive degradation of 1,3-dinitrobenzene. Additionally, Favaretto et al.^24^ reported that phenolic acids are predominant among the polyphenol constituents of the Poaceae family, which includes carpet grass. Given the lack of commercially available standard solutions for these phenolic acids, further in-depth investigation is required in this area. Ion chromatography analysis conducted throughout the reaction process identified nitrite as an intermediate byproduct, which eventually diminished. The quantification of both nitrate and nitrite was carried out and used for kinetic analysis. While this analysis focused on these specific compounds, identifying and quantifying all other potential byproducts is crucial to ensure no harmful substances are produced. Therefore, employing additional analytical techniques may be necessary in future studies.

As seen in Figure S7, effluent DO dropped below 1 mg/L and remained at this low level until the end of the experiment. The low DO levels were likely caused by the reaction between polyphenol released from the carpet grass and oxygen, which consumed the DO.^29^ EC increased from 220 μS/cm to 430 μS/cm at 24 hs, peaked at 660 μS/cm at 48 h, and then gradually decreased to 280 μS/cm by the end of the experiment, a trend similar to that of polyphenols. pH remained slightly acidic between 5.3 and 6.0. ORP dropped below 100 mV between 10 and 24 h, then fluctuated between 50 and 160 mV, indicating a reducing state.

## 4 Conclusion

Surface water runoff containing nitrate is a common environmental concern. Reducing nitrate levels in surface water runoff and surface water bodies can help prevent nitrate infiltration into subsurface groundwater. This study hypothesized that when nitrate-laden surface water runoff flows over land covered with carpet grass vegetation, it comes into contact with natural polyphenol reductants released by the grass. This interaction leads to the denitrification of nitrate to nitrogen gas, thereby purifying the water. The results indicate that polyphenol concentrations in aqueous solutions ranging from 20 to 80 mg-GAE/L, with a retention time of 24 h, are effective in sequentially reducing nitrate to nitrite and eventually to nitrogen gas. The reaction kinetics were thoroughly examined, and the associated rate constants were determined. Using natural vegetation, such as carpet grass, for nitrate reduction offers an environmentally friendly in situ alternative to conventional chemical treatments typically applied in above-ground treatment units, making it applicable in contaminated water management practices.

## Supporting Information Available

Gibbs free energy for selected compounds (Table S1); the dimension of simulated field application flow tank and calculation of flow retention time (Table S2); the experimental matrix design for exploring the effect of weed dose and flow rate on nitrate removal rate (Table S3); the derivations of the equation for NO_2_^–^ reaction kinetics (Table S4); the variation of water qualities during the flow of NO_3_^–^ solution with 30 g of grass at retention time of 8 h (Figure S1); the variation of water qualities during the flow of NO_3_^–^ solution with 100 g of grass at retention time of 8 h (Figure S2); the variation of water qualities during the flow of NO_3_^–^ solution with 30 g of grass at retention time of 24 h (Figure S3); the variation of water qualities during the flow of NO_3_^–^ solution with 100 g of grass at retention time of 24 h (Figure S4); the variation of water qualities during the flow of NO_3_^–^ solution without grass at retention time of 24 h (control test 1) (Figure S5); the variation of water qualities during the flow of water with 100 g of grass at retention time of 24 h (control test 2) (Figure S6); the variation of water qualities during the flow of NO_3_^–^ solution with 100 g of grass at retention time of 24 h under an extended reaction time (Figure S7) (PDF)
