# Supplementary material for: Carpet Grass Polyphenol Reductive Degradation of Aqueous Nitrate—A Conceptual Field Application Study
Source: ACS Omega. 2024 Nov 14;9(47):46943–9. doi: 10.1021/acsomega.4c06522 (PMC11603230; doi:10.1021/acsomega.4c06522)
Supplement: Supplementary file 1 — ao4c06522_si_001.pdf [file ao4c06522_si_001.pdf]

Supplementary Materials

Carpet grass polyphenol reductive degradation of aqueous nitrate – A conceptual field application study

Chenju Liang[*,1], Wei-Sin Tao[1], Chia-Lu Shih[1], Yi-Wun Ye[1], Chun-Ting Li[1], Chi-Wei Wang[2]

[1] Department of Environmental Engineering, National Chung Hsing University

145 Xingda Road, Taichung 402, Taiwan

[2] Department of Environmental Engineering, Da-Yeh University

168 University Road, Dacun, Changhua 515006, Taiwan

*Corresponding author. Tel.: +886-4-22856610; Fax: +886-4-22856610

E-mail address: cliang@nchu.edu.tw

Table S1. Gibbs free energy for selected compounds in equations (1) to (3).

	$\Delta_r G$, in kJ/mol	References
$Ph(OH)_2 \rightarrow Ph(OH)O^- + H^+$ (proton affinity)	-32.73	[1] Eq. (16)
$Ph(OH)O^- \rightarrow Ph(OH)O^{\cdot} + e^-$ (electron transfer enthalpy)	8.65	[1] Eq. (17)
NO_3^-	-111.34	[2]
N_2	0	[2]
H_2O	-237.18	[2]

Table S2. The dimension of simulated field application flow tank and calculation of flow retention time.

When fixed slope of 5°, the water mark of the inner tank as picture shown (unit: cm). 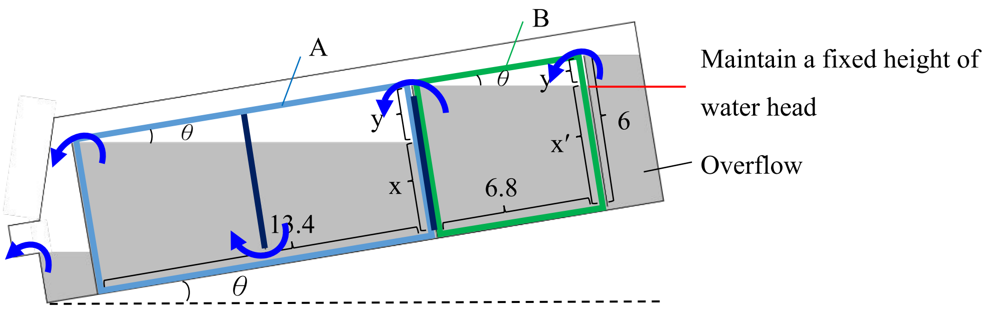	
The specification of tank (A + B)	The length, width and height of the reaction tank (A+B): 21 cm, 14 cm, and 6 cm, respectively.
Water contained in tank A with tank slop θ = 5°	$\tan 5° = \frac{y}{13.4}$, $y = 1.2$, $x = 6 - y = 4.8$<br>Water volume $= \frac{13.4\times(4.8+6)}{2} \times 14 = 1013$ cm^3
Water contained in tank B with tank slop θ = 5°	Same as above, $y' = 0.6$, $x' = 5.4$<br>Water volume $= 543$ cm^3
Total water volume	$A + B = 1013 + 543 ≒ 1560$ cm^3
Retention time (containing 30 g weeds)	Volumes of 30 g weeds: approximately 40 cm^3,<br>Water volume (containing 30 g weeds) $=$<br>$1560 - 40 = 1520$ cm^3
	When the outflow rate is 1 mL/min,<br>Retention time $= 1520/1 = 1520$ min $= 25$ h
	When the outflow rate is 3 mL/min,<br>Retention time $= 1520/3 = 507$ min $= 9$ h
Retention time (containing 100 g weeds)	Volume of 100 g weeds: approximately 150 cm^3<br>Same as above, the retention time of the tank containing 100 g weeds is 24 h, 8 h

Table S3. The experimental matrix design for exploring the effect of weed dose and flow rate on nitrate removal rate.

Group	NO_3^- inflow (mg/L)	Grass dose (g)	Retention time [a] (h)	Outflow rate [b] (mL/min)	Sampling time (h)	Parameters measured
1	110	30	8	3	0、8、24	NO_3^-<br>NO_2^-<br>Polyphenol<br>ORP<br>DO<br>EC<br>pH
2		100				
3		30	24	1	0、24、48	
4		100			0、24、48、72	
Control 1		0				
Control 2	0	100				

[a] The retention time calculated by considering the volume of weeds and tank that can be accommodated at fixed slope of 5°.

[b] The outflow rate obtained by adjusting the inflow rate and the tilt angle of the tank.

Table S4. The derivations of the equation for NO_2^- reaction kinetics and the application of Newton's method to solve for k_2.

$\frac{d[NO_2^-]_t}{dt} = \alpha k_{obs,\ NO_3^-}[NO_3^-] - k_2[NO_2^-]_t$<br>$= \alpha k_{obs,\ NO_3^-}[NO_3^-]_0 e^{-k_{obs,\ NO_3^-}t} - k_2[NO_2^-]_t$
Using the ordinary differential equation to obtain the integrating factor:<br>$y' + p(x)y = Q(x)$<br>$\rightarrow \frac{d[NO_2^-]_t}{dt} + k_2[NO_2^-]_t = \alpha k_{obs,\ NO_3^-}[NO_3^-]_0 e^{-k_{obs,\ NO_3^-}t}$<br><br>$u(x) = e^{\int p(x)dx}$<br>$\rightarrow u(t) = e^{\int k_2 dt} = e^{k_2 t}$<br><br>$y = \frac{1}{u(x)}\int Q(x)u(x)dx$<br>$\rightarrow [NO_2^-]_t = \frac{1}{e^{k_2 t}}\int \alpha k_{obs,\ NO_3^-}[NO_3^-]_0 e^{-k_{obs,\ NO_3^-}t} e^{k_2 t}dt$<br>$= \frac{\alpha k_{obs,\ NO_3^-}[NO_3^-]_0}{e^{k_2 t}}\int_0^t e^{(k_2 - k_{obs,\ NO_3^-})t}dt$<br>$= \frac{\alpha k_{obs,\ NO_3^-}[NO_3^-]_0}{e^{k_2 t}} \times \frac{1}{k_2 - k_{obs,\ NO_3^-}} e^{(k_2 - k_{obs,\ NO_3^-})t} \Big\|_0^t$<br>$= \frac{k_{obs,\ NO_3^-}[NO_2^-]_{max}}{(k_2 - k_{obs,\ NO_3^-})e^{k_2 t}}(e^{(k_2 - k_{obs,\ NO_3^-})t} - e^{(k_2 - k_{obs,\ NO_3^-})0})$<br>$= \frac{k_{obs,\ NO_3^-}[NO_2^-]_{max}}{(k_2 - k_{obs,\ NO_3^-})e^{k_2 t}}(e^{(k_2 - k_{obs,\ NO_3^-})t} - 1)$<br>$= \frac{k_{obs,\ NO_3^-}[NO_2^-]_{max}}{k_2 - k_{obs,\ NO_3^-}}(e^{-k_{obs,\ NO_3^-}t} - e^{-k_2 t})$
$[NO_2^-]_t = \frac{k_{obs,\ NO_3^-}[NO_2^-]_{max}}{k_2 - k_{obs,\ NO_3^-}}(e^{-k_{obs,\ NO_3^-}t} - e^{-k_2 t})$
Expanding the above equation:

$$\rightarrow \frac{[NO_2^-]_t}{[NO_2^-]_{max}} = \frac{k_{obs,\ NO_3^-}}{k_2 - k_{obs,\ NO_3^-}}(e^{-k_{obs,\ NO_3^-}t} - e^{-k_2 t})$$

$$= \frac{k_{obs,\ NO_3^-}\ e^{-k_{obs,\ NO_3^-}t}}{k_2 - k_{obs,\ NO_3^-}} - \frac{k_{obs,\ NO_3^-}\ e^{-k_2 t}}{k_2 - k_{obs,\ NO_3^-}}$$

$$\rightarrow (k_2 - k_{obs,\ NO_3^-})\frac{[NO_2^-]_t}{[NO_2^-]_{max}} = k_{obs,\ NO_3^-}e^{-k_{obs,\ NO_3^-}t} - k_{obs,\ NO_3^-}e^{-k_2 t}$$

$$\rightarrow k_2\frac{[NO_2^-]_t}{[NO_2^-]_{max}} - k_{obs,\ NO_3^-}\frac{[NO_2^-]_t}{[NO_2^-]_{max}} = k_{obs,\ NO_3^-}\ e^{-k_{obs,\ NO_3^-}t} - k_{obs,\ NO_3^-}\ e^{-k_2 t}$$

Let $\frac{[NO_2^-]_t}{[NO_2^-]_{max}} = A$ and $k_2 = x$

$$\rightarrow Ax + k_{obs,\ NO_3^-}e^{-tx} - k_{obs,\ NO_3^-}A - k_{obs,\ NO_3^-}e^{-k_{obs,\ NO_3^-}t} = 0$$

$$\rightarrow Ax + k_{obs,\ NO_3^-}\ e^{-tx} - \left(k_{obs,\ NO_3^-}A + k_{obs,\ NO_3^-}e^{-k_{obs,\ NO_3^-}t}\right) = 0$$

The k_2 of the above equation is solved by Newton's method:

$$f(x) = AX + k_{obs,\ NO_3^-}e^{-tx} - (k_{obs,\ NO_3^-}A + k_{obs,\ NO_3^-}e^{-k_{obs,\ NO_3^-}t})$$

$$f'(x) = -tk_{obs,\ NO_3^-}e^{-tx} + A$$

Using the Newton’s method to solve the k_2 of equation in excel.

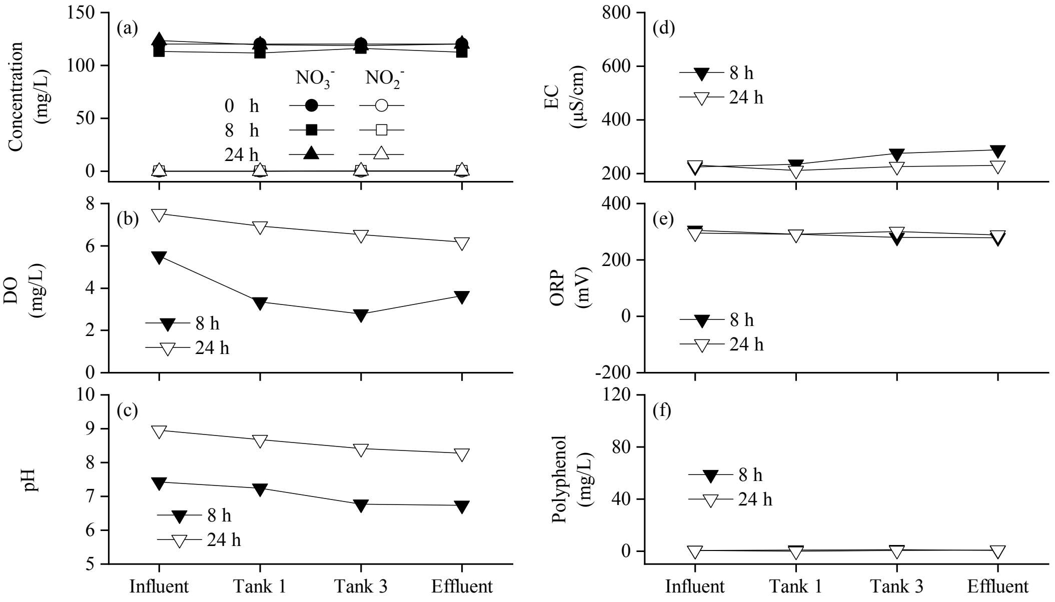

Figure S1. The variation of water qualities (a) NO_3^- and NO_2^-, (b) DO, (c) pH, (d) EC, (e) ORP, and (f) polyphenol during the flow of NO_3^- solution at Inflow, Tank 1, Tank 3, and Outflow sampling points, with 30 g of grass at retention time of 8 h.

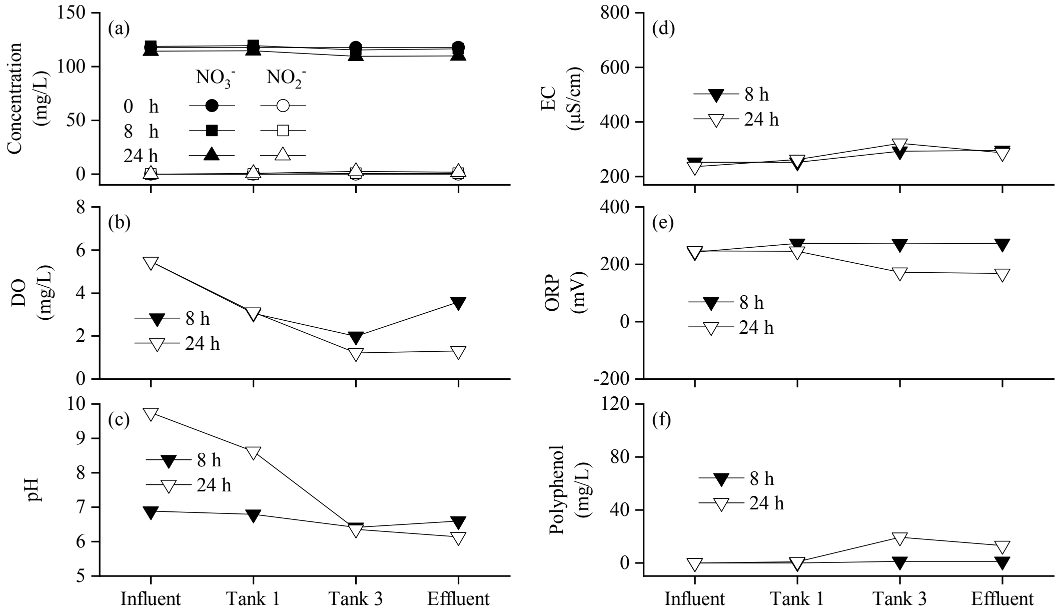

Figure S2. The variation of water qualities (a) NO_3^-, and NO_2^-, (b) DO, (c) pH, (d) EC, (e) ORP, and (f) polyphenol during the flow of NO_3^- solution at Inflow, Tank 1, Tank 3, and Outflow sampling points, with 100 g of grass at retention time of 8 h.

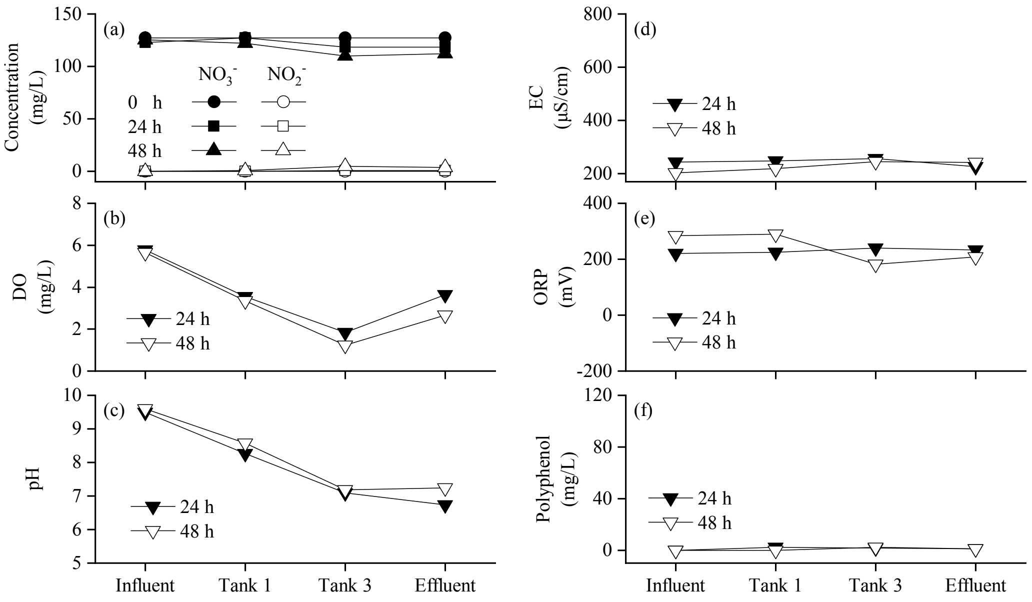

Figure S3. The variation of water qualities (a) NO_3^- and NO_2^-, (b) DO, (c) pH, (d) EC, (e) ORP, and (f) polyphenol during the flow of NO_3^- solution at Inflow, Tank 1, Tank 3, and Outflow sampling points, with 30 g of grass at retention time of 24 h.

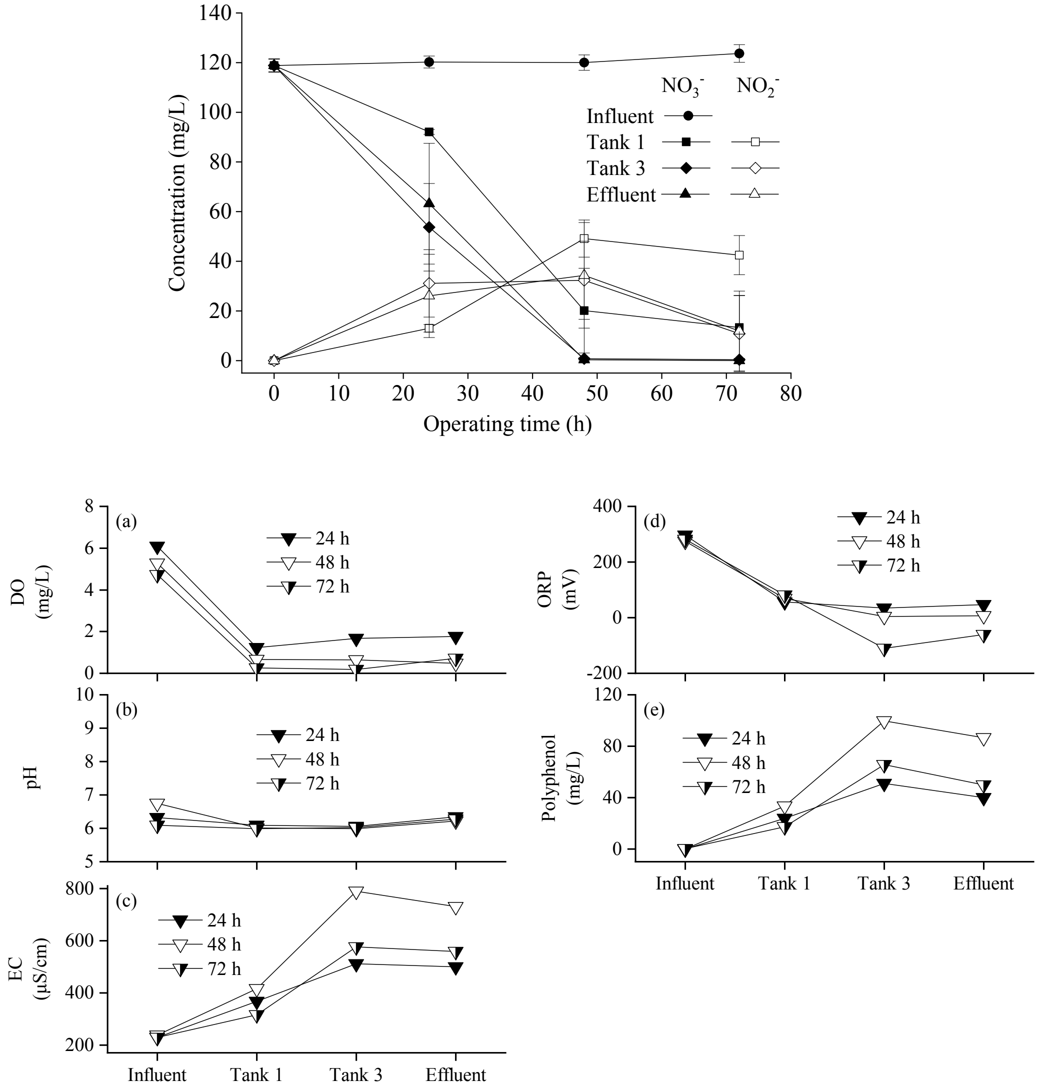

Figure S4. The variation of water qualities (a) NO_3^- and NO_2^-, (b) DO, (c) pH, (d) EC, (e) ORP, and (f) Polyphenol during the flow of NO_3^- solution at Inflow, Tank 1, Tank 3, and Outflow sampling points, with 100 g of grass at retention time of 24 h.

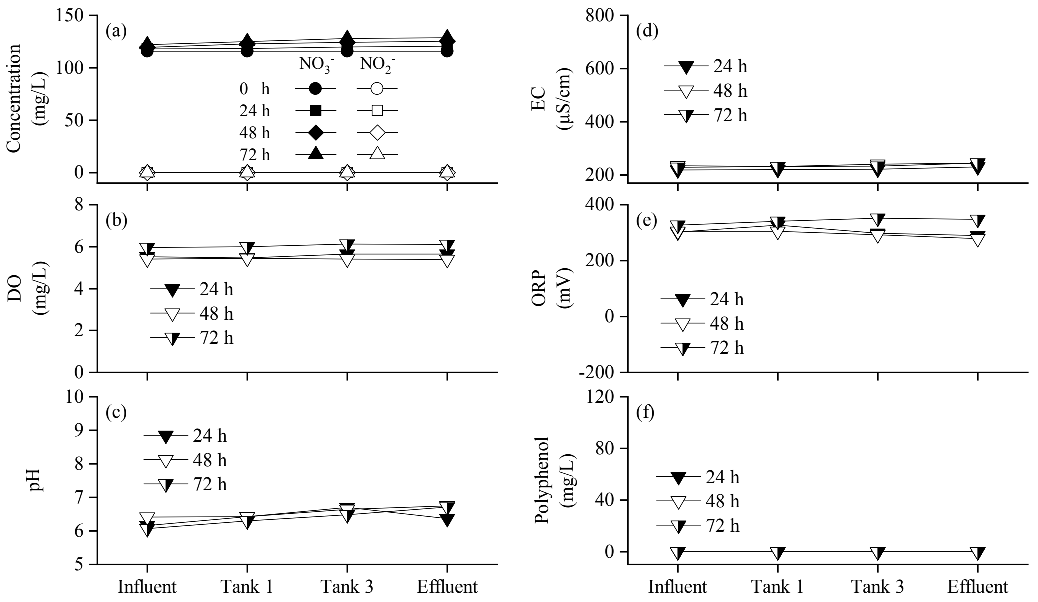

Figure S5. The variation of water qualities (a) NO_3^- and NO_2^-, (b) DO, (c) pH, (d) EC, (e) ORP, and (f) Polyphenol during the flow of NO_3^- solution at Inflow, Tank 1, Tank 3, and Outflow sampling points, without grass at retention time of 24 h (control test 1).

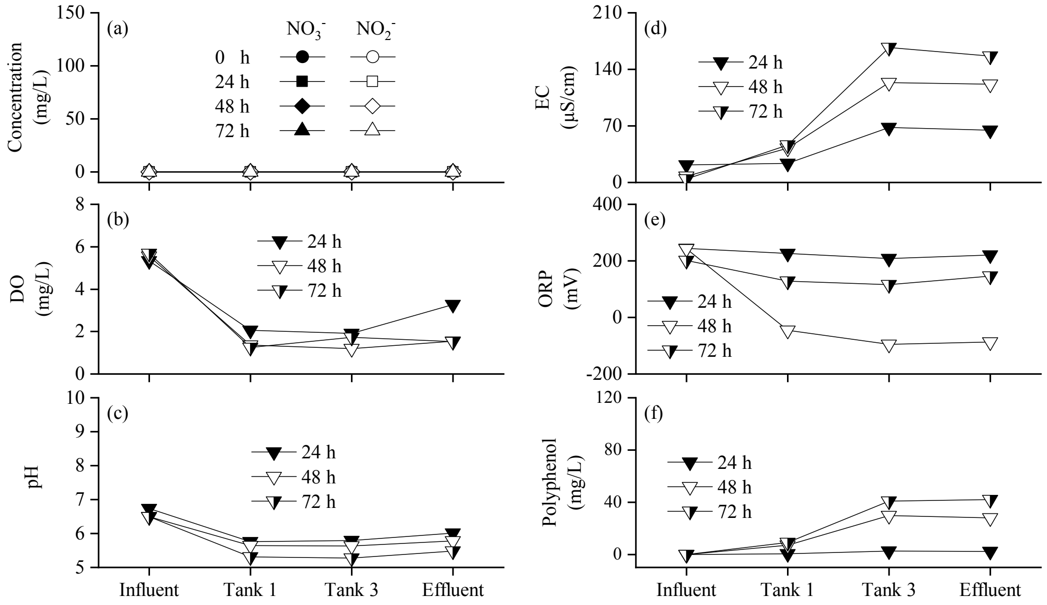

Figure S6. The variation of water qualities (a) NO_3^- and NO_2^-, (b) DO, (c) pH, (d) EC, (e) ORP, and (f) Polyphenol during the flow of solution without NO_3^-, at Inflow, Tank 1, Tank 3, and Outflow sampling points, with 100 g of grass at retention time of 24 h (control test 2).

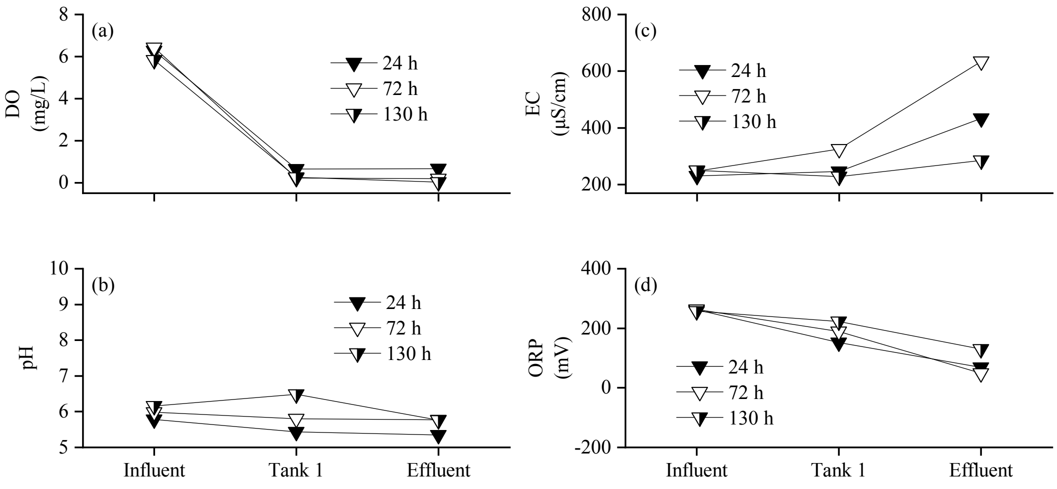

Figure S7. The variation of water qualities (a) NO_3^- and NO_2^-, (b) DO, (c) pH, (d) EC, (e) ORP, and (f) Polyphenol during the flow of NO_3^- solution at Inflow, Tank 1, and Outflow sampling points, with 100 g of grass at retention time of 24 h under an extended reaction time.

References:

[1] A. Amić, B. Lučić, V. Stepanić, Z. Marković, S. Marković, J.M. Dimitrić Marković, D. Amić, Free radical scavenging potency of quercetin catecholic colonic metabolites: Thermodynamics of $2H^+/2e^-$ processes, Food Chemistry, 218 (2017) 144-151.

[2] C. Sawyer, P. McCarty, G. Parkin, Chemistry for Environmental Engineering and Science, McGraw-Hill Education, New York, 2003.
